# Temperature and humidity index (THI) affects salivary cortisol (HC) and dehydroepiandrosterone (DHEA) concentrations in growing bulls following stress generated by performance test procedures

**DOI:** 10.3389/fvets.2023.1237634

**Published:** 2023-07-25

**Authors:** Elisa Giaretta, Paolo Mongillo, Laura Da Dalt, Matteo Gianesella, Martina Bortoletti, Lorenzo Degano, Daniele Vicario, Gianfranco Gabai

**Affiliations:** ^1^Department of Comparative Biomedicine and Food Science, University of Padua, Legnaro, Italy; ^2^Department of Animal Medicine, Production and Health, University of Padua, Legnaro, Italy; ^3^Associazione Nazionale Allevatori Bovini di Razza Pezzata Rossa Italiana (A.N.A.P.R.I.), Udine, Italy

**Keywords:** biomarkers, cortisol, DHEA, growing bulls, habituation, saliva, stress, THI

## Abstract

The hypothalamus-pituitary–adrenal axis response to a challenge was proposed for genetic selection of robust and resilient animals. As ACTH (adrenocorticotropic hormone) test and hormone measurements in blood may result impractical, it may be useful to measure salivary hormones in response to natural stressors, after an accurate biological validation, to control factors that could contribute to the response. We evaluated whether animal handling during performance test affects salivary HC and DHEA secretion and could be used for selection. We tested the effects of habituation to repeated handling and THI as putative bias. Bull calves (*N* = 273) undergoing performance test were sampled at 8–9 and 11–13 months (*N* = 101), 8–9 months (*N* = 131), or 11–13 months (*N* = 41). On each test day (D0), calves were isolated, conducted to a squeeze chute and immobilized for 6 min. Saliva samples were collected in the morning after feed administration (T0), and after 6 min immobilization in the squeeze chute (T1) for HC and DHEA measurement. Environmental temperature and relative humidity were recorded every hour from 1:00 h to 24:00 h during the 6 days before the performance test and on D0. Salivary HC and DHEA concentrations were higher in T1 (*p* < 0.01), although a clear individual positive response to handling could be observed in less than 10% of subjects. The mixed model revealed: (i) HC and HC/DHEA were higher in Young bulls (*p* < 0.05). (ii) The time of T0 sample collection significantly affected DHEA (*p* < 0.01) and HC/DHEA (*p* < 0.05). (iii) THI affected both steroids (*p* < 0.001) but not HC/DHEA. Spearman correlations suggested that THI weakly affected salivary HC at T0 only (*ρ* = 0.150, *p* < 0.01), while moderate statistically significant correlations were found between DHEA and THI at T0 (*ρ* = 0.316, *p* < 0.001), and T1 (*ρ* = 0.353, *p* < 0.001). Salivary HC and DHEA in response to handling procedures might identify subpopulations of subjects with sensitive HPA axis. Habituation to repeated handling played a role, as the hormone response was lower in older animals. Chronic exposure to high THI had a minor effect on salivary HC visible at T0. A more intense THI effect was observed on salivary DHEA concentrations at both T0 and T1, which should be worth of further investigations.

## 1. Introduction

Glucocorticoid hormones are implicated in a number of biological actions on metabolism, cardiovascular system, inflammatory processes and brain functions. The hypothalamus-pituitary-adrenocortical (HPA) axis is central in the regulation of energy metabolism and stress response, and it is activated when an animal perceives a real or presumptive threat. In farm animals, the definition of stress mostly relies on glucocorticoid (in mammals: cortisol or hydrocortisone, HC) measurement (1).

The level of the HPA axis activity influences production traits negatively and some robustness traits positively and, in modern high producing animals, genetic selection for production traits has probably contributed to the reduction of HPA axis activity and, consequently, robustness (2). For this reason, monitoring of the HPA axis activity has been proposed as a tool for modern selection programs, which consider the combination of different breeding goals, such as high production potentials, low sensitivity to environmental perturbations (robustness), and the capacity of animals to recover quickly from environmental challenges (resilience) (3, 4). Temperament is another trait that is object of animal selection, as quieter and calmer animals during handling have greater individual performances than excitable ones (5), which can also be dangerous to personnel. Moreover, easier animal handling can influence the human-animal relationship and improve animal welfare. In cattle, plasma HC is related to animal temperament, as it is correlated to variables such as exit velocity (rate at which cattle exit a squeeze chute and traversed a fixed distance) and pen scores (score ascertained from animal behavior while penned in small groups) (6, 7).

In pigs, the adrenal response to an ACTH challenge is highly heritable. In divergent selection experiments, Large White pigs were classified as high and low ACTH responders, based on the cortisol concentrations measured in blood 1 h after ACTH administration (8). In the second-generation offspring, high ACTH responders seemed more capable to respond to acute social stress and display increased resistance to pathogens, and an overall less pronounced stress effects (9). Higher lymphocyte counts and TNFα secretion were confirmed in the third generation of high ACTH responders, which display also a greater oxygen-carrying capacity (10). Conversely, in cattle, despite the use of ACTH challenge is considered as appropriate to investigate the HPA axis (6), we are not aware of studies aiming at assessing the heritability of the HPA response to ACTH and exploring the possibility to genetically select for HPA responsiveness. A recent work explored the repeatability of the ACTH response in growing bulls, which is a relevant issue when performing studies that allocate cattle to groups of low and high HC responders (11). Results of that study did not fully support the hypothesis that salivary HC responsiveness to ACTH in fattening bulls is repeatable, and suggest that the effect of the physiological state can have a profound influence on ACTH responsiveness in these animals.

When testing a large number of animals is the objective, the ACTH test followed by hormone measurements in blood can be impractical for both technical and ethical reasons. Under those circumstances, the biological response to a natural stressor or to an elicited stressor, such as animal handling while performing a growth performance test, may fulfill the purpose of testing the HPA reactivity. This could be even more useful if hormones could be measured non-invasively in saliva instead of blood. This procedure, however, requires an accurate biological validation, which implies the quantification of the stressor magnitude and the control of other factors that could contribute to the response (12).

Dehydroepiandrosterone (DHEA) and its sulfate (DHEAS) may help in drawing a more precise picture of the HPA activity and stress response (13). The mammal adrenals can synthesize both DHEA and DHEAS, even though the adrenal contribution to the amount of these steroids in the circulation is species-specific and, at least in humans, their secretion is affected by stress (14). In addition, although the functions of these steroids are not fully elucidated, available data obtained in humans and laboratory animals suggest that they can counteract the effects of glucocorticoid hormones, mostly by their anti-aging, immune enhancing and neuroprotective properties (13, 14).

The HPA axis is a key factor in the adaptation to thermal stress and, therefore, the effects of environmental temperature and humidity should be carefully assessed when studying the physiological response to stressors. In most vertebrates, both the increase and decrease in environmental temperature are generally associated with increased HC concentrations, in particular during short exposure. Therefore, changes in temperature are perceived as a stressor by most animals. However, many animals may be able to adapt to prolonged variations in temperature, provided the changes are not extreme and fall within the range of temperatures experienced in their natural environment (15). In beef calves, blood HC concentrations increased following the exposure to severe heat stress and returned to the normal range after 9 days, suggesting that calves can maintain homeostasis during the long-term heat stress (16). In Holstein bull calves, saliva HC increased during acute heat stress exposure and followed the changes in THI with higher levels during the daytime (17). Conversely, Ronchi et al. (18) observed a decrease in plasma HC concentrations in pubertal heifers chronically exposed to high air temperatures. To the best of our knowledge, the effects of changes in environmental temperature on DHEA secretion have not been studied so far. For these reasons, a deeper knowledge of the secretion patterns of this steroid in blood and saliva represent an important aspect to investigate.

To explore the possibility to use salivary HC and DHEA as a tool for selecting robust and/or non-temperamental animals, we hypothesized that handling procedures related to growth performance test, such as catching and immobilization with head restraint in a squeeze chute, could represent a stressor for growing bulls, which could affect HC and DHEA concentrations in saliva. As biomarkers should be submitted to biological validation before being used for their purpose (1, 12), in this study, other factors that could contribute to the salivary HC and DHEA responses were controlled. In particular, as the procedures are repeated throughout the year on a large number of subjects, this was a good opportunity for studying the effects of age and environmental temperature and humidity, distilled in the concept of Temperature/Humidity Index (THI), as a putative bias affecting the salivary hormone response and the HC/DHEA molar ratio.

## 2. Materials and methods

### 2.1. Farm and management

The study was performed at the Genetic Centre of the Italian Simmental Breeder Association (ANAPRI) located in Fiume Veneto, northeast Italy (45.9250° N, 12.7323° E). The Italian Simmental is a dual-purpose breed, and every year approximately 200 male calves are tested for their growth performance in order to select animals destined for breeding. Male calves born from programmed breeding are collected from ANAPRI associates and arrive at the Genetic Centre at the age of approximately 30 days. Upon arrivals, animals are quarantined in a designated barn at about 1 km from the Centre, and are weaned at the age of 4 months. Then, animals are transferred in indoor stall-slatted units and are allocated in pens of 5–6 individuals (3.4–4.0 m^2^/head). The stalls are not provided with artificial cooling system. After an adaptation period of about 30 days, the growth performance test begins. The test terminates when animals are 12–13 month old. During this period, animals undergo body weight and morphometric characteristics measurement every 4–6 weeks.

During the whole testing period, calves have free access to water and received *ad libitum* a total mixed-ration (TMR), distributed once a day between 7:30 and 9:00 h, and balanced to meet their nutritional requirements and in line with the beef feeding system in use in northern Italy (19). The TMR was based on ground corn (2.9 kg DM), corn silage (2.8 kg DM), sunflower and rapeseed meals (1.5 kg DM), wheat straw (0.9 kg DM), barley (0.6 kg DM), dried beet pulp (0.6 kg DM), wheat bran (0.6 kg DM), soybean meal (0.2 kg DM), and a mineral and vitamin mix supplement (0.2 kg DM), which contained vitamin A (100,000 IU/kg), vitamin D3 (12,000 IU/kg), vitamin E (450 mg/kg), choline chloride (1,000 mg/kg), FeCO_3_ (1,076 mg/kg), KI (39 mg/kg), Ca(IO_3_)_2_ (21.6 mg/kg), Mn_2_O_3_ (1,161 mg/kg), CuSO_4_•5H_2_O (275 mg/kg), ZnO (620 mg/kg), ZnSO_4_ (2055 mg/kg), Na_2_SeO_3_ (3.1 mg/kg), urea (50,000 mg/kg), and *Saccharomyces cerevisiae* MUCL 39,885 (120 × 109 CFU/kg) (20).

### 2.2. Experimental protocol

Experimental procedures were planned in order to generate the least modifications to activities routinely performed during the performance test. The study was performed between May 2018 and September 2020, and consisted of 20 test days (Table 1). On each test day (D0), calves were isolated, conducted from the pen to a squeeze chute equipped with weighting and head restraint systems and immobilized for 6 min. During this time, calves were measured for frame growth including hip height, hip width, heart girth, paunch girth, and body weight. The whole operations were performed by the same skilled operators, and lasted 8–10 min from the isolation to the release of the calf. In the context of this experiment, the whole procedures were considered as the stressor.

**Table 1:** Bull calves enrolled in the experiment grouped by age (Young: 8–9 month-old; Old: 11–13 month-old), and Temperature Humidity Index (THI) observed on the test day (D0) and during the 6 days before the test day (D-6, D-1).

ID	Test day (D0)	Calves (N)		Observations recorded between D-6 and D0				THI values observed from 21:00 h to 7:00 h during the night before sampling				
		Young	Old	No HS	Mild HS	Severe HS	AuC (THI*h)	Mode^a^	Mean	Median	Min	Max
S01	03/05/2018	18	–	121	28	19	10,921	64.5	65.6	65.4	64.5	67.3
S02	06/06/2018	17	–	95	48	25	11,984	64.8	63.9	62.4	59.5	72.7
S03	05/07/2018	18	–	84	43	41	12,266	69.1	74.3	70.8	66.2	85.5
S04	02/08/2018	16	–	31	60	77	13,209	60.7	76.2	76.3	73.5	78.2
S05	06/09/2019	–	16	111	46	11	11,650	64.2	65.4	64.9	61.5	73.8
S06	05/10/2018	16	11	154	5	9	9,578	42.1	50.5	50.0	48.0	57.8
S07	09/11/2018	17	15	163	5	0	9,633	53.2	52.4	52.3	49.5	55.2
S08	11/12/2018	12	16	168	0	0	6,734	31.3	29.8	30.0	26.9	32.8
S09	11/01/2019	7	17	168	0	0	5,115	14.4	22.4	23.7	16.3	26.6
S10	08/02/2019	12	17	168	0	0	6,959	41.4	36.6	37.5	30.5	39.5
S11	15/03/2019	18	11	166	2	0	7,526	24.6	32.0	32.7	26.4	39.6
S12	06/09/2019	15	–	113	19	36	11,232	48.7	61.6	63.9	54.8	65.4
S13	04/10/2019	11	–	132	13	23	10,422	58.3	40.9	41.0	35.8	52.1
S14	08/11/2019	7	–	168	0	0	8,563	50.4	47.9	49.8	41.0	50.4
S15	06/12/2019	15	–	168	0	0	6,335	22.5	32.7	36.1	22.5	39.4
S16	14/01/2020	16	–	168	0	0	5,662	20.3	22.1	21.9	18.1	26.4
S17	06/02/2020	16	–	168	0	0	6,880	44.6	35.5	36.4	25.1	45.1
S18	04/06/2020	–	17	123	20	25	10,665	54.0	59.2	58.1	55.0	68.3
S19	08/07/2020	–	7	91	26	51	11,965	58.5	58.2	56.3	53.6	71.4
S20	04/09/2020	–	17	118	31	19	11,257	57.9	57.6	56.7	54.1	64.9

THI data were recorded every hour from 1:00 h of D-6 to 24:00 h of D0 (*N* = 168 observations).

THI = 0.8 × T + RH/100 × (T−14.4) + 46.4; T, environmental temperature (°C); RH, relative humidity (%); HS, heat stress.

No HS, No risk of Heat Stress, THI < 72; Mild HS, Mild risk of Heat Stress, 72 ≤ THI < 79; Severe HS, Severe risk of Heat Stress, THI ≥ 79.

^a^When the distribution is multimodal, the lower mode value is reported.

Overall, the study involved 273 bull calves belonging to two age groups: Young calves (8–9 month old) and Old calves (11–13 month old). In particular, 101 animals were sampled both at the age of 8–9 and 11–13 months, 131 animals were sampled at the age of 8–9 months only, and 41 calves were sampled at the age of 11–13 months only (Table 1), totalizing 375 observations. All the animals involved in the trial had already experienced the performance test procedures at least twice at the date of their first sampling.

Saliva samples were collected using a swab (Salivettes, Sarstedt, Nümbrecht, Germany) in the morning after TMR administration (T0), and immediately before the releasing from the squeeze chute (T1). The swab held by surgical forceps was introduced in the calf’s mouth, which was allowed to chew for 30 s. At least 1 mL saliva was collected by this procedure. The T0 samples were collected between 9:30 and 14:00 h, while the T1 samples were collected between after 12:30 h.

Environmental temperature and relative humidity data were collected every hour from 1:00 h to 24:00 h during the 6 days (D-6, D-1) before D0 and on D0. Data were obtained from a weather station of the Regional Agency for Environment Protection (ARPA Friuli Venezia Giulia),^1^ located at 11 km from the Genetic Centre (45.895661° N, 12.814989° E). THI was calculated by the formula: THI = 0.8 × T + RH/100 × (T−14.4) + 46.4.

### 2.3. Hormone analyses

HC and DHEA concentrations in saliva samples (50 μL) were analyzed in duplicates by solid-phase microtiter RIAs (21, 22). For the HC RIA, the intra- and inter-assay coefficients of variation (CV) were 3.1 and 12.0%, respectively. For the DHEA RIA, the intra- and inter-assay CV were 6.4 and 13.9%, respectively. The RIA methods were validated for bovine saliva by parallelism and recovery tests.

To assess parallelism, saliva samples (*N* = 3) were serially diluted (1:2–1:32) in assay buffer (PBS 0.01 M, 0.1% BSA, pH 7.2). Recovery tests were performed by adding known amounts of hormones (120–1,000 pg./mL) to saliva samples (*N* = 3) and calculating the regression curves obtained between the observed and expected hormone concentrations.

### 2.4. Statistical analyses

All statistical analyses were performed using the software IBM SPSS Statistics (version 28.0). The level of significance was set at *p* < 0.05.

To study the parallelism between the calibration and the sample dilution curves, the regression curves between the ratios of the bound hormone fractions and the bound at zero hormone concentration (B/B0) and the 10-logarithm of hormone concentrations, were calculated for both the sample dilution and calibration curves. Then, the slopes of regression curves were compared by analysis of covariance. Recovery tests were expressed as the regression curves between 10-log transformed observed and expected hormone concentrations. A slope not statistically different from 1 indicated that salivary components do not interfere with assay accuracy.

The Kolmogorov–Smirnov test was used to compare hormone distributions observed at T0 and T1. To estimate the number of animals that responded to the growth performance test procedures, four types of response were arbitrarily determined: negative (DT1-T0 ≤ 0), uncertain (0 < DT1-T0 < Q3T0 and HT1 < Q3T0), feeble (0 < DT1-T0 < Q3T0 and HT1 ≥ Q3T0) and positive (DT1-T0 ≥ Q3T0 and HT1 ≥ Q3T0), where:

- DT1-T0: Difference in hormone concentration (T1 – T0);
- HT1: Hormone concentration measured at T1;
- Q3T0: 75th percentile of the distribution of hormone concentrations measured at T0.

The T1-T0 differences in hormone concentrations could be influenced by the time of sample collection at T0, as it is known that hormones concentrations, in particular HC, display circadian variations (23). For this reason, the effect of the time of sampling at T0 was investigated in HC, DHEA and their molar ratio. Hormone concentrations observed at T0 were grouped in three Sampling Time (ST) intervals (ST9: 9:00–9:59 h, ST10: 10:00–10:59 h, ST11: after 11:00 h), and their distributions were compared by Kruskal-Wallis’s ANOVA.

Hormone concentrations in response to growth performance testing were analyzed by a mixed model for analysis of variance, with the Calf within Sampling Day as random factors. The model included the fixed effects:

- Sample (S; T0, T1);
- Age (A; Young: 8–9 month-old; Old: 11–13 month-old);
- THI recorded at the time of sampling (No risk of Heat Stress: THI < 72; Mild risk of Heat Stress: 72 ≤ THI < 79; Severe risk of Heat Stress: THI ≥ 79);
- Sampling Time at T0 (ST9, ST10 and ST11);
- The interactions S*A, S*THI, S*ST.

Salivary HC and DHEA concentrations and their molar ratio are reported as estimated marginal means (± s.e.m.). Pairwise comparisons of main effects and their interactions were performed using the EMMEANS subcommand with Bonferroni adjustment.

The impact of a single THI value on the distress experienced by the animals may be less informative than a figure representing annoying THI values over a time interval. For this reason, the impact of THI on the concentrations of HC and DHEA were also explored by studying the Spearman’s correlations between hormone concentrations measured at T0 and T1 and the following parameters representing the THI exposure:

- the THI values observed on the day of sampling (D0) and between 21:00 h and 7:00 h during the night before sampling (mean, median, min, max values);
- the number of observations with No Heat Stress (THI < 72); Mild Heat Stress (72 ≤ THI < 79) and Severe Heat Stress (THI ≥ 79) recorded between D-6 and D0;
- the area under the curve (AuC, THI*hr) calculated between D-6 and D0 by the linear trapezoidal method;
- the mode value for THI recorded between D-6 and D0.

## 3. Results

### 3.1. RIAs validation

Data of the validation tests of HC and DHEA RIAs are shown in Table 2. Both assays displayed a good degree of parallelism, indicating that hormone concentrations could be detected across a wide range of dilutions. The DHEA method was characterized by an acceptable degree of recovery that ranged between 90.2 and 113.5%, and the slope of the regression curve between the 10-log transformed observed and expected hormone concentrations was not significantly different from 1. Conversely, the HC RIAs was characterized by a slight underestimation, as recovery ranged between 86.2 and 110.3%.

**Table 2:** Validation of the RIA methods for the detection of HC and DHEA in saliva samples of bull calves.

		Cortisol (HC)		Dehydroepiandrosterone (DHEA)	
		Standard curve (*N* = 3)	Saliva samples (*N* = 3)	Standard curve (*N* = 3)	Saliva samples (*N* = 3)
Parallelism tests	Linear regression	B/B_0_ = 104.4–43.41*Log[HC]	B/B_0_ = 103.8–43.13*Log[HC]	B/B_0_ = 93.2–39.71*Log[DHEA]	B/B_0_ = 86.1–36.62*Log[DHEA]
	*R* ^2^	0.985	0.980	0.989	0.980
	Analyses of covariance	*F* = 0.034; *p* = 0.855	*F* = 1.422; *p* = 0.239		
Recovery tests	Linear regression		[HC]_obs_ = 0.9 + 0.92*[HC]_exp_		[DHEA]_obs_ = 3.7 + 0.96*[DHEA]_exp_
	*R* ^2^		0.998		0.986
	Analyses of covariance		*F* = 63.789; *p* = 0.001		*F* = 2.928; *p* = 0.094

Parallelism was assessed between the assay standard curves and the serial dilutions of saliva samples in assay buffer. The slopes of the linear regression curves obtained for the standard curve and saliva sample dilutions were compared by analysis of covariance. Recovery was assessed by adding known amounts of hormones to saliva samples, and was expressed as the regression curves between observed (obs) and expected (exp) hormone concentrations [C] expressed in pg./well. The slopes of the recovery regression curves were compared with the bisector curve by analysis of covariance.

### 3.2. Statistic distributions of raw hormone concentrations

When rough data were analyzed, both salivary HC and DHEA concentrations showed significantly different distributions between samples collected at T0 and T1 (*p* < 0.001; Table 3), and means were higher in T1 for both hormones. No differences between T0 and T1 were found in the distribution of the HC/DHEA molar ratio.

**Table 3:** Distribution of cortisol (HC), dehydroepiandrosterone (DHEA) concentrations, the HC/DHEA molar ratio, and type of individual response (T0 vs. T1).

									Individual response (N)			
Distribution									Negative	Uncertain	Feeble	Positive
Hormone	Sample	Mean ± s.e.m.	Min	Q1	Q2	Q3	Max	Kolmogorov–Smirnov test	D_T1-T0_ ≤ 0	0 < D_T1-T0_ < Q3_T0_ H_T1_ < Q3_T0_	0 < _DT1-T0_ < Q3_T0_ H_T1_ ≥ Q3_T0_	D_T1-T0_ ≥ Q3_T0_ H_T1_:≥ Q3_T0_
HC (nM)	T0	0.77 ± 0.02	0.13	0.54	0.70	0.88	4.81	*D* = 2.702 *P* < 0.001	141	86	114	34
	T1	1.01 ± 0.05	0.16	0.58	0.81	1.11	10.39					
DHEA (nM)	T0	3.58 ± 0.10	0.35	2.02	3.15	4.63	12.99	*D* = 2.337 *P* < 0.001	117	121	106	31
	T1	4.64 ± 0.14	0.65	2.57	3.92	6.26	14.61					
HC/DHEA	T0	0.27 ± 0.01	0.04	0.15	0.22	0.33	2.32	*D* = 1.059 *p* < 0.212	226	117	0	32
	T1	0.33 ± 0.05	0.03	0.13	0.19	0.31	13.19					

HC and DHEA concentrations were measured in saliva samples of growing bulls (*N* = 375) collected in the morning (T0) and after the growth performance test procedures, immediately before the releasing from the squeeze chute (T1). The distributions of hormone concentrations were compared by the Kolmogorov–Smirnov test (IBM SPSS 28.01). Q1, 25th percentile; Q2, 50th percentile (median); Q3, 75th percentile; D_T1-T0_, Difference in hormone concentration (T1 – T0); H_T1_, Hormone concentration at T1; Q3_T0_, 75th percentile of the distribution of hormone concentrations measured at T0.

Despite the differences in distributions, a clear positive increase in T1 was observed in 34 (HC) and 31 (DHEA) cases only (Table 3).

**Figure 1:**
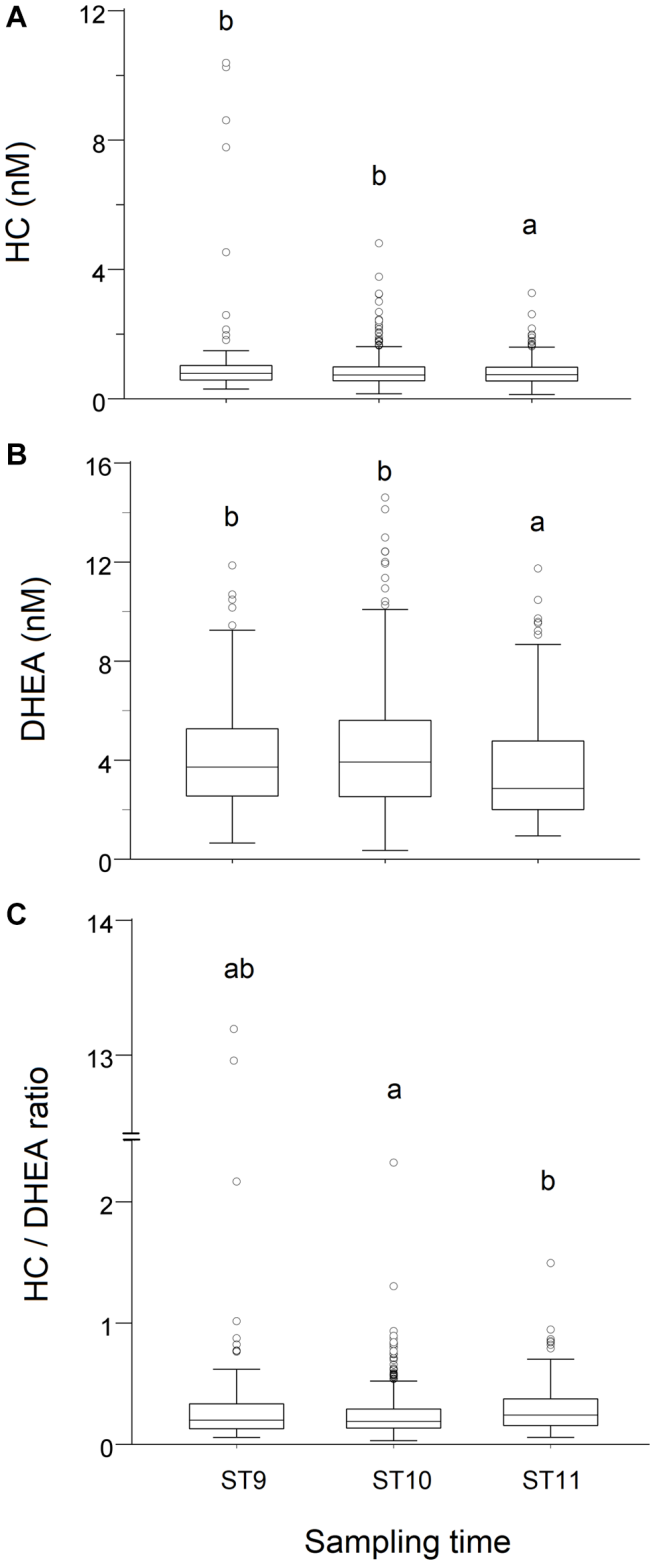
Effect of the time of sampling on the distribution of cortisol (HC, **A**) and dehydroepiandrosterone (DHEA, **B**) concentrations, and on the HC/DHEA ratio **(C)**, measured in saliva samples of growing bulls collected in the morning (T0). Observations were grouped in three Sampling Time (ST) intervals (ST9: 9:00–9:59 h, ST10: 10:00–10:59 h, ST11: after 11 h). Different superscripts indicate significantly different data distributions (*p* < 0.01; Kruskal-Wallis’s ANOVA—IBM SPSS 28.01).

The concentrations of both steroids measured in T0 were significantly higher (*p* < 0.01) in samples collected before 11:00 h, whereas the HC/DHEA molar ratio was significantly higher (*p* < 0.01) in samples collected after 11 h (Figure 1).

### 3.3. Factors affecting HC and DHEA concentrations

**Figure 2:**
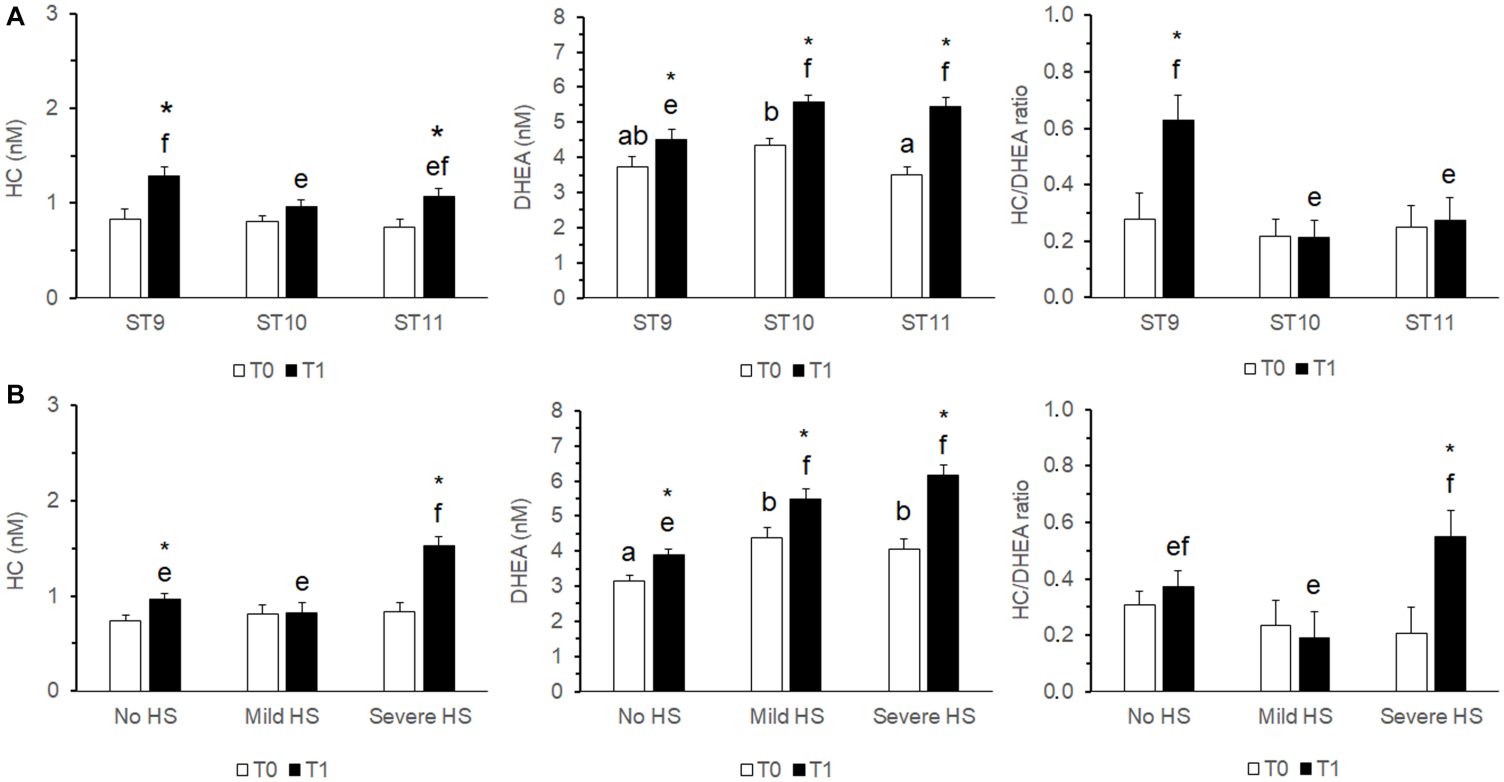
Estimated marginal means (± s.e.m.) of the cortisol (HC), dehydroepiandrosterone (DHEA) concentrations and their molar ratio measured in saliva samples of growing bulls (*N* = 375) collected in the morning (T0) and after the growth performance test procedures immediately before the releasing from the squeeze chute (T1). The effects of S*ST **(A)** and S*THI **(B)** are shown. Different superscripts above the bars indicate significantly different means (^a,b^ T0; ^e,f^ T1; *p* < 0.05). The asterisk above the T1 bar indicates a significant difference between T0 and T1 (*p < 0.05). S: Sample (T0, T1). THI, temperature-humidity index recorded at the time of sampling (No HS, No risk of Heat Stress, THI < 72; Mild HS, Mild risk of Heat Stress, 72 ≤ THI < 79; Severe HS, Severe risk of Heat Stress, THI ≥ 79). ST, Sampling Time: time intervals when the morning (T0) samples were collected (ST9: 9:00–9:59 h, ST10: 10:00–10:59 h, ST11: after 11:00 h).

The outcomes of the mixed model analysis of variance are shown in Table 4. The variability explained by the fixed factors was 9, 14.8, and 4.5% for HC, DHEA and HC/DHEA, respectively (Nagakawa’s marginal pseudo R-square coefficient of determination). A highly significant effect of Sample (S; *p* < 0.001) was observed for both HC and DHEA concentrations, which were significantly higher at T1 than T0. HC concentrations and the HC/DHEA molar ratio were significantly higher in Young bulls (*p* < 0.05). Significant effects of THI and S*THI (*p* < 0.01) were observed for HC and DHEA. The Sampling Time (ST) significantly affected DHEA concentrations (*p* < 0.01) and the HC/DHEA molar ratio (*p* < 0.05), while the S*ST interaction affected the DHEA concentrations (*p* < 0.05) only. The estimated marginal means showing the S*THI and S*ST effects are displayed in Figure 2.

**Table 4:** Mixed model analysis of variance (F and P) of the cortisol (HC), dehydroepiandrosterone (DHEA) concentrations and their molar ratio.

Fixed factors	HC		DHEA		HC/DHEA	
	*F*	*P*	*F*	*P*	*F*	*P*
Intercept	682.24	<0.001	1426.57	<0.001	87.75	<0.001
Sample (S)	19.99	<0.001	59.27	<0.001	3.68	0.056
Age (A)	5.99	0.015	0.860	0.355	4.80	0.029
THI	7.28	<0.001	29.83	<0.001	1.96	0.142
Sampling Time (ST)	2.00	0.136	5.53	0.004	4.36	0.013
S * A	1.01	0.315	1.18	0.278	2.28	0.134
S * THI	5.59	0.004	5.50	0.004	2.20	0.102
S * ST	1.98	0.140	5.05	0.007	2.70	0.069
Nakagawa’s pseudo-R Square coefficients of determination						
Marginal	0.090		0.148		0.045	
Conditional	0.115		0.438		0.077	

Hormones concentrations were measured in saliva samples of growing bulls (*N* = 375) collected in the morning (T0) and after the growth performance test procedures immediately before the releasing from the squeeze chute (T1). Sample, T0, T1; Age, Young (8–9 month-old), Old (11–13 month-old); THI, temperature-humidity index recorded at the time of sampling (No Heat Stress: THI < 72; Mild Heat Stress: 72 ≤ THI < 79; Severe Heat Stress: THI ≥ 79); Sampling Time, time of morning (T0) samples collection (ST9: 9:00–9:59 h, ST10: 10:00–10:59 h, ST11: after 11:00 h).

Correlations between hormone concentrations and THI parameters were studied by the Spearman’s rho and they are shown in Table 5. Salivary HC concentrations measured at T0 showed weak statistically significant correlations with most parameters describing the THI impact. Conversely, no correlations were found between HC concentrations at T1 and all THI parameters. Moderate statistically significant correlations were found between DHEA measured at both T0 and T1 and all the THI parameters. Interestingly, the stronger correlations were found between salivary DHEA and those parameters describing the chronicity of high THI exposure (AuC, mode).

**Table 5:** Correlations (Spearman’s rho; *N* = 375) between THI parameters and cortisol (HC) and dehydroepiandrosterone (DHEA) concentrations measured in saliva samples of growing bulls collected in the morning (T0) and after the growth performance test procedures.

			HC		DHEA	
			T0	T1	T0	T1
THI values observed at sampling		Rho	0.150	0.041	0.316	0.353
		*P*	0.004	0.430	<0.001	<0.001
Number of observations recorded between D-6 and D0	No HS (THI < 72)	Rho	−0.147	−0.037	−0.422	−0.380
		*P*	0.004	0.480	<0.001	<0.001
	Mild HS 72 ≤ THI < 79	Rho	0.148	0.028	0.435	0.416
		*P*	0.004	0.585	<0.001	<0.001
	Severe HS THI ≥ 79	Rho	0.081	0.013	0.299	0.200
		*P*	0.117	0.804	<0.001	<0.001
	Mild + Severe HS	Rho	0.147	0.037	0.422	0.380
		*P*	0.004	0.480	<0.001	<0.001
	AuC (THI*hr)	Rho	0.143	0.027	0.432	0.416
		*P*	0.005	0.598	<0.001	<0.001
	Mode (THI values)	Rho	0.081	0.017	0.437	0.365
		*P*	0.117	0.736	<0.001	<0.001
THI values observed between 21:00 h and 7:00 h during the night before sampling	Median	Rho	0.087	0.050	0.364	0.382
		*P*	0.093	0.330	<0.001	<0.001
	Min	Rho	0.113	0.055	0.435	0.410
		*P*	0.028	0.285	<0.001	<0.001
	Max	Rho	0.134	0.031	0.373	0.356
		*P*	0.010	0.546	<0.001	<0.001

Immediately before the releasing from the squeeze chute (T1). THI, Temperature Humidity Index; HS, Heat Stress; AuC, area under the curve.

## 4. Discussion

Testing the pituitary response to an ACTH challenge can be an impractical tool for selecting robust and/or non-temperamental animals, in particular if the procedure involves a large number of young bulls. For this reason, we explored the possibility to use salivary HC and DHEA and submitted these putative biomarkers to biological validation (1, 12). We had the opportunity to examine the HPA response to a growth performance test, without interfering with the test procedures routinely adopted in the field. The procedures consisted in the animal separation from conspecifics, contact with people and restraint in a squeeze chute. Indeed, restraint, contact with people and exposure to novelty are psychological stressors that could be considered as a mild to strong stressor, to which animals can display variable responses depending to their individual sensitivity to the stressor itself (24). It is also hypothesizable that the HPA axis response reflects the animal sensitivity to the applied stressor.

Among the factors that can affect the response of salivary steroids to the procedures adopted in this experiment, we could check for the effects of age (A), the time of the T0 sample collection (ST) and the environmental temperature and relative humidity (THI). These variables, however, explained a low degree of the total variability observed for both salivary steroids, as suggested by the marginal Nakagawa’s pseudo-R square coefficients of determination.

Genetic factors contribute to determine how fearful an animal may become when it is handled (25). It is conceivable that animals fearing interactions with humans more (more temperamental subjects) display higher HC concentrations than calmer subjects do. Functional characteristics of the HPA axis varied with temperament in Brahman beef heifers, whose temperament was evaluated by exit velocity measurement. In particular, excitable heifers showed higher stress responsiveness to handling, increased basal adrenal function (excitable animals had higher HC) but not increased basal pituitary function (ACTH was not different between calm and temperamental subjects), and an attenuated adrenal response to ACTH (6).

The HC and DHEA concentrations in saliva samples after the performance test procedures (T1) were compared with those observed in the morning in absence of visible stressors (T0). Salivary HC and DHEA concentrations measured at T0 were on average lower than those measured at T1. However, when considering the individual responses, they were negative (hormone concentrations were lower in T1 than in T0) or likely attributable to the daily physiological fluctuations of these steroids (responses classified as uncertain or feeble) in a large number of subjects. Certainly, we are aware that the distribution of the individual responses depends upon the classification criteria adopted, which were arbitrarily defined in order to obtain a sufficiently conservative definition of a “positive” response. Following our definition, less than 10% of tests resulted in a “positive” stress response, in term of HC concentrations observed at T1.

It is possible that the performance test procedures were too mild as a stressor to induce an increase in HC secretion detectable in saliva. As the test was repeated every 4–6 weeks, and animals enrolled in this experiment had experienced the procedures at least twice at the time of our first measurement, an effect of their previous experiences cannot be excluded. Previous experiences can have a considerable impact on the stress response. Cattle trained and habituated to a squeeze chute may have baseline cortisol levels and be behaviorally calmer than not trained animals that may also display higher cortisol levels (24). As an example, no differences in both serum and hair cortisol were found between excitable and calm young Angus bulls subjected to performance testing when the young bulls enrolled in the study have had already experienced the performance test procedures at least three times before the experiment, which could have led to acclimation (7) or behavioral habituation (26). Also temperamental heifers become calmer and can acclimate to frequent handling (27). Accordingly, the observation that in this experiment HC concentrations and the HC/DHEA molar ratio were significantly higher in younger bulls could be partially explained by animals’ acclimation to the test procedures, which are repeated throughout their first year of life.

The overall duration of the whole procedures, from bull-calf isolation from the herd mates to its release from the squeeze chute, could have been too short to elicit a visible salivary HC response. In cattle, salivary HC reflects the free hormone fraction measured in plasma (28), with a delay of approximately 10 min of the salivary in comparison with the plasma HC peak following a stressful procedure (29). However, basal salivary HC concentrations are poorly correlated to those found in blood during feeding and drinking procedures (30), and it is possible that only intense stress or pain, mimicked by an ATCH challenge, can affect salivary HC (1). Basal salivary HC concentrations observed in this experiment are comparable with those measured in newborn calves (31), fattening bulls (11), and dairy cows (28–30), even though many samples fell in the lower portion of the concentration ranges reported in those papers. This last finding can be explained, at least in part, by a slight underestimation of HC concentrations by our RIA method, as suggested by the recovery test.

Although salivary DHEA concentrations were higher at T1 and, therefore, a positive effect of the performance test procedures could be hypothesized, a clear positive response was observed in about 8% of cases only. For this reason, it is difficult to ascribe the increase in salivary DHEA to the stressor represented by testing procedures. In non-primate mammals, the effect of stress on DHEA release is still controversial as, often, factors that can affect DHEA release other than stress (e.g., inflammatory and reproductive status, or the chronicity of exposure to stressors) are not carefully considered (14). Salivary DHEA concentrations found in this work are comparable to those found by Aleman et al. (31) that, to the best of our knowledge, published the only one paper reporting on salivary DHEA concentrations in the bovine. Those authors investigated the concentrations of several steroids in serum and saliva in newborn heifer-calves from two to 48 h after birth, and found that DHEA concentrations in saliva were higher than in serum (31). Unfortunately, in our work, it was not possible to compare serum and salivary DHEA and the origin of salivary DHEA can be object of speculation only.

In cattle, HC secretion shows a temporal correlation with the light–dark cycle, with higher HC levels recorded in the morning at the onset of daylight (23). The distribution of the rough data observed at T0 for salivary HC in this study agrees with the circadian secretory patterns observed in bulls and, on average, they were higher in samples collected before 11:00 h. We are not aware of studies that investigated the circadian secretion of DHEA in cattle. Studies in humans have demonstrated a circadian pattern of DHEA secretions in young subjects, which tend to disappear with aging (32, 33). Notably, in humans, serum and salivary DHEA showed an elevated degree of correlation, and an elevated decline in salivary DHEA can be observed between 8 and 12 a.m. (33). Patterns of salivary DHEA concentrations at T0 observed in this study support the hypothesis of a daily rhythmicity of DHEA secretion also in the bovine. With those premises, we tested if the time at T0 sample (ST) can affect the response of both steroids to the performance test procedures. We could observe a significant effect of ST and S*ST interaction for the DHEA response. In particular, the salivary DHEA response was greater when the T0 saliva samples were collected after 10:00 h. Conversely, such an effect was not observed for salivary HC, even though pairwise comparisons of the S*ST interaction suggested a stronger HC release at T1 when the T0 samples were collected before 10:00 h. A clear explanation of these observations is not possible based on our experimental setting. However, these findings suggested that the time of sampling should be carefully considered in order to correctly interpret the hormone response to a stressor.

Environmental characteristics are important modulators of the animals’ endocrine system and, in general, they should be taken into deep consideration when investigating endocrine responses. In particular, the stress response can be affected by environmental temperature and humidity, which are known stimulators of the HPA axis (15–17). The temperature-humidity index (THI) has been effectively used as an indicator of heat stress, and environmental conditions consisting of high temperature and relative humidity negatively affect cattle physiology, and often lead to heat stress, reduced performance and decreased animal comfort (34). In our study, THI was calculated using the formula used by Mader et al. (34). In the genetic station, bull calves were housed indoor without artificial cooling system. Outdoor THI values can represent the environmental conditions to which animals were exposed, even though they may not exactly reflect the indoor microclimate experiencing by the animals; in particular, it should be consider that the bull-calves were not exposed to solar radiation. Under our experimental setting, the effects of THI on salivary HC and DHEA concentrations were quite different.

The correlation observed between salivary HC concentrations measured at T0 and the parameters describing THI exposure, in particular the area under the curve describing the THI during 7 days before sampling, were very low, even though significant. Conversely, no significant correlations between salivary HC concentration measured at T1 and the same THI parameters were observed, and this is suggestive of a poor effect of THI on the HPA axis under our experimental conditions. Nevertheless, it is important to consider that we tested the effects of THI on the HPA response during the 7 days before sampling. During those time intervals, THI never reached extreme values and conditions of severe heat stress were rarely observed. In beef calves, increased plasma HC levels were observed after rapid exposure to severe heat stress (THI: 88–90) but, if heat stress conditions were maintained, plasma HC levels returned to the normal range after 9 days implying that adaptation could be occurred (16). Kovács et al. (17) observed that saliva HC increased during acute heat stress exposure and followed the changes in THI with higher levels during the daytime in pre-weaned Holstein bull calves. In that study, however, THI significantly decrease during nighttime possibly leading to HPA recoveries of calves from heat stress, followed by lower daytime cortisol release. These observations support the hypothesis that calves, as many vertebrates, can adapt to prolonged variations in temperature, provided the changes are not extreme (15). Likely, in this study salivary HC concentrations were measured in animals that were already accustomed to environmental temperature and humidity in both cold and hot weather. Under these circumstances, it is possible that management or animal handling is still a major stimulus for HC release. However, the possibility that the HPA response is blunted due to high THI cannot be discarded, and further investigations on the chronic environmental effects on the HPA response should be performed in the bovine.

To the best of our knowledge, this study documented for the first time a positive relationship between THI and salivary DHEA secretion, and it is noteworthy that this relationship was evident at both T0 and T1, suggesting that temperature and relative humidity exerted an additive effect on the stress-induced increase in salivary DHEA.

Whether the DHEA increase originates from the adrenals in response to increased THI cannot be ruled out in this experiment. The findings by Aleman et al. (31) that DHEA concentrations are higher in saliva than plasma, however, raise the possibility that the salivary glands could be a source of DHEA. Indeed, this may reflect the presence/activation of steroidogenic enzymes, or sulfatase enzyme, which converts the sulfated form of DHEA (DHEAS) into DHEA (14). Conversely, despite saliva flow rate varies during the day and it is affected by stimuli related to feeding (35), the salivary concentration of HC and other unconjugated steroids is not affected by saliva flow rates, and it is the result of passive diffusion of free steroids, HC in particular, across the acinar cells of the salivary gland (12).

Mammalian salivary glands own the machinery for metabolizing steroid hormones. The submaxillary salivary gland of mature and immature domestic pigs can metabolize DHEA into androstenedione, 5-alpha-androstane-3,17-dione and androsterone, and to small amounts of testosterone, 5-alpha-dihydrotestosterone and 5-alpha-androstanediols (36). More recently, it was observed that rat salivary gland homogenates could synthesize corticosterone and testosterone from pregnenolone, but not pregnenolone from cholesterol (37). Those authors hypothesized that the precursor of steroidogenesis in the rat salivary gland is pregnenolone-sulfate, which is abundant in the rat circulation. The presence of steroidogenic enzymes capable to metabolize DHEA (3beta- and 17beta-hydroxysteroid dehydrogenases, in particular) was observed also in the salivary glands of healthy humans (38). Interestingly, the presence of steroid sulfatase and sulfotransferase enzymes, responsible, respectively, for the sulfonation and desulfation reactions of steroid hormones, was also observed (38). To the best of our knowledge, no study is available on the expression of steroidogenic enzymes in cattle salivary glands. However, DHEA-S is present in the bovine circulation at nanomolar levels (39) and, therefore, it is conceivable that the salivary glands can uptake the circulating DHEA-S and convert it into DHEA.

Despite the relationship between increasing THI and salivary DHEA secretion still deserves a thorough investigation, experimental evidences support the hypothesis that DHEA is implicated in the regulation of body temperature. In fact, dietary administration of DHEA-acetate increased resting heat production in rats (40), and, in mice, parenteral DHEA administration induces hypothermia in a dose-dependent manner and independently of its ability to cause food restriction, to affect serotonin or dopamine functions (41), or to act via its downstream steroid metabolites.

In conclusion, handling procedures of the growth performance test did not represent a stressor sufficient to stimulate a consistent response of salivary HC or DHEA in most growing bulls. However, a response to handling could be observed at least in a number of young bulls, despite its intensity was often low. For this reason, the hypothesis the measurement of salivary HC and DHEA in response to handling procedures may be a tool to identify subpopulations of subjects with more sensitive HPA axis cannot be entirely discarded. Nevertheless, the heritability of the HC response (difference between salivary HC measured in T1 and T0) was estimated in a limited number of young bulls and was on average lower that 20% (unpublished data).

It is important to bear in mind that several factors can affect salivary hormone secretion. For example, habituation to repeated handling may have played a role, as the hormone response was lower in older animals. In addition, the chronic exposure to high, even if not extreme, environmental temperature and humidity (THI) had only a minor effect on salivary HC that was visible in the morning sample (T0). Conversely, a more intense effect of THI was observed on salivary DHEA concentrations at both T0 and T1, which should be worth of further investigations.

## Data availability statement

The raw data supporting the conclusions of this article will be made available by the authors, without undue reservation.

## Ethics statement

The animal study was reviewed and approved by the Authority in Charge for Animal Welfare of the University of Padua.

## Author contributions

GG, PM, and DV conceived and designed the study. DV acquired the funding. PM, EG, GG, and MG managed data acquisition and sample collection and drafted sections of the manuscript. EG, LDD, and MB performed the hormone assays. LD and EG organized the database and performed the statistical analysis. GG, EG, and PM wrote the manuscript. All authors revised and approved the final version of the manuscript.

## Funding

This work was supported by the project “Dual Breeding” (National Rural Development Program -PSRN 2014/2020 – sub-measure 10.2), funded by the Italian Ministry of Agriculture.

## Conflict of interest

The authors declare that the research was conducted in the absence of any commercial or financial relationships that could be construed as a potential conflict of interest.

## Publisher’s note

All claims expressed in this article are solely those of the authors and do not necessarily represent those of their affiliated organizations, or those of the publisher, the editors and the reviewers. Any product that may be evaluated in this article, or claim that may be made by its manufacturer, is not guaranteed or endorsed by the publisher.

## Footnotes

^1^ https://www.meteo.fvg.it
